# Development of SNAP-Tag Based Nanobodies as Secondary Antibody Mimics for Indirect Immunofluorescence Assays

**DOI:** 10.3390/cells14100691

**Published:** 2025-05-10

**Authors:** Wenjie Sheng, Chaoyu Zhang, T. M. Mohiuddin, Marwah Al-Rawe, Roland Schmitz, Marcus Niebert, Lutz Konrad, Steffen Wagner, Felix Zeppernick, Ivo Meinhold-Heerlein, Ahmad Fawzi Hussain

**Affiliations:** 1 Department of Gynecology and Obstetrics, Medical Faculty, Justus-Liebig-University Giessen, Klinikstr. 33, 35392 Giessen, Germany; wenjie.sheng@med.uni-giessen.de (W.S.); zhangchaoyu1993@126.com (C.Z.); tm.mohiuddin@mhb-fontane.de (T.M.M.); lutz.konrad@gyn.med.uni-giessen.de (L.K.); felix.zeppernick@gyn.med.uni-giessen.de (F.Z.); ivo.meinhold-heerlein@gyn.med.uni-giessen.de (I.M.-H.); 2 Clinic for Gynecology and Obstetrics, University Hospital Brandenburg, Medizinische Hochschule Brandenburg Campus GmbH, Hochstraße 29, 14770 Brandenburg an der Havel, Germany; 3 Institute of Pathology, University Hospital Giessen, Justus-Liebig-University Giessen, Langhanssstr. 10, 35392 Giessen, Germany; roland.schmitz@patho.med.uni-giessen.de (R.S.); marcus.niebert@web.de (M.N.); 4 Department of Otorhinolaryngology, Head and Neck Surgery, University of Giessen, 35392 Giessen, Germany; steffen.wagner@hno.med.uni-giessen.de

**Keywords:** immunofluorescence assays, fluorescence imaging, nanobody, SNAP-tag technology, secondary antibody

## Abstract

The immunofluorescence assay is widely used for cellular biology and diagnosis applications. Such an antigen–antibody detection system enables the assessment and visualization of the expression and localization of target proteins. In the classical indirect immunofluorescence assay, secondary antibodies are conjugated to fluorophores. However, conventional secondary antibodies have limited applications due to their large size (150 kDa). Moreover, as animal-derived products, secondary antibodies are associated with ethical concerns and batch-to-batch variability. In this study, we developed fluorescence-labeled recombinant nanobodies as secondary antibodies by utilizing previously established anti–mouse and anti–rabbit IgG secondary nanobodies in combination with the self-labeling SNAP-tag. Nanobodies, which are significantly smaller (15 kDa), are capable to detect primary antibodies produced in mice and rabbits. The SNAP-tag (20 kDa) enables site-specific binding of various O^6^-benzylguanine (BG)-modified fluorophores to the recombinant nanobodies. These recombinant nanobodies were produced using mammalian cell expression system, and their specific binding to mouse or rabbit antibodies was validated using flow cytometry and multi-color fluorescence microscopy. The low cost, easy of expression, purification and site-specific conjugation procedures for these anti–mouse and anti–rabbit IgG secondary nanobodies make them an attractive alternative to traditional secondary antibodies for indirect immunofluorescence assays.

## 1. Introduction

Fluorescence probes are powerful tools for the detection of proteins and other molecules, widely applied in biomedical research to visualize molecule localization, gene expression, protein function and tumor cell progression [1,2,3]. Importantly, fluorescence technology enables non-invasive imaging in vivo making it a cornerstone of modern biological imaging.

Fluorescent proteins (FPs), such as green fluorescent protein (GFP), are conventional tools in this field. By fusing FPs to a protein of interest (POI), researchers can visually track the localization and cellular activity of the POI [4,5,6]. However, FPs come with notable limitations, including relatively large size, weak photostability and narrow spectral range compared to small organic dyes. These limitations have hindered broader applications of FPs [7]. To address these challenges, self-labeling proteins have emerged as an effective alternative, enabling the specific labeling of POI with organic dyes or small molecules. Among these, the SNAP-tag has gained significant attention. The SNAP-tag is a 20 kDa protein derived from a mutant form of the human repair protein O^6^-alkylguanine-DNA alkyltransferase (hAGT). This tag reacts specifically with substrate containing O^6^-benzylguanine (BG) [8], forming an irreversible covalent bond between the BG-modified molecule and the POI [9,10]. The SNAP-tag offers a fast and highly specific conjugation method, with numerous commercially available BG substrates (e.g., BG-GLA-NHS, BG-biotin, BG-fluorophores) [11,12,13]. These substrates expand the versatility of synthetic probes, enabling diverse applications in biological imaging and beyond.

The nanobody (Nb), as the smallest natural antibody fragment with complete antigen-binding capacity and derived from camelids, is another appealing molecule. Nb is made up of a single variable heavy chain (VHH) with a molecular weight of only 15 kDa, which represents only 1/10 the size of regular antibodies [14]. Furthermore, Nb has high solubility and binding-affinity [15,16], and Nb’s unique complementarity determining regions (CDRs) structure offers a larger antigen-interacting surface. Therefore, Nb can detect hidden or non-accessible sites that conventional antibodies are unable to recognize [17,18]. In light of the advantages of Nb, an increasing number of strategies for diagnosis and treatment were introduced [19,20].

The immunofluorescence assay is a powerful tool for detecting antigens in cells and tissues, offered in two formats: direct or indirect. The indirect immunofluorescence assay (IFA) is particularly advantageous because it utilizes a two-step process involving primary antibodies and secondary antibodies, where the secondary antibody has usually conjugated with fluorophores to amplify the signal. This approach provides higher sensitivity, stronger fluorescence signals, and greater flexibility in staining compared to the direct method, where the primary antibody is conjugated with a fluorophore. Primary antibodies, predominantly derived from mice and rabbits, are essential for both basic research and clinical research. Secondary antibodies, such as anti-mouse IgG and anti-rabbit IgG antibodies, their production process traditionally includes immunizing host animals (goats, donkeys or sheep), harvesting and purifying the serum, which not only raises ethical concerns due to the high number of animals required but also leads to variability among production batches, reducing the reproducibility of the experimental results.

To address these challenges, many researchers advocate for reduced- or non-animal methods of antibody production [21,22,23]. Nb, with their smaller size, high specificity, and ease of engineering, present a promising alternative. Leveraging these advantages, a toolbox of anti-mouse IgG and anti-rabbit IgG Nbs was developed. These Nbs were conjugated to maleimide dyes via engineered cysteines and subsequently applied in immunofluorescence assays. This innovative approach not only addresses the ethical concerns and reproducibility issues associated with traditional secondary antibody production but also enhances the versatility and reliability of immunofluorescence techniques [24].

In this study, we selected five anti-mouse IgG and anti-rabbit IgG Nbs [24], and fused them with the self-labeling protein, SNAP-tag. This fusion enabled the site-specific conjugation of various fluorophores, providing a flexible and efficient labeling strategy. The performance of these Nb-based constructs in IFA was thoroughly investigated and evaluated. This approach presents a promising alternative to traditional secondary antibody production methods, offering several key advantages. It significantly reduces the reliance on animals, addressing ethical concerns while maintaining high specificity and reproducibility. Moreover, this method is relatively cost-effective and feasible on a laboratory scale, making it a practical solution for the further development of animal-reduced research techniques in immunofluorescence applications.

## 2. Materials and Methods

### 2.1. Cell Culture

The breast cancer cell lines MDA-MB-468 (ACC 738), MDA-MB-231 (ACC 732), MDA-MB-453 (ACC 65), Hs578T (ACC 781) and MCF-7 (ACC 115) were maintained in DMEM (Gibco, Schwerte, Germany) supplemented with 10% fetal calf serum (FBS) and 1% penicillin-streptomycin (complete DMEM). The endometrial cell lines (kindly provided by Lutz Konrad) 49Z and 12Z were also cultured in complete DMEM medium. T-HESC cells were cultured in DMEM/F12 (Gibco, Schwerte, Germany) supplemented with 10% FBS, 1% penicillin-streptomycin and 1% insulin-transferrin-selenium-ethanolamine (ITS-X, 100×; ThermoFischer Scientific, Schwerte, Germany). Ishikawa cells were cultured in MEM (Biowest, Nuaillé, France), supplemented with 5% FBS (Gibco, Schwerte, Germany) and 1% non-essential amino acids (NEAA; Gibco, Schwerte, Germany). The human embryonic kidney line HEK293T was cultured in RPMI 1640 (Gibco, Schwerte, Germany) containing 10% FBS and 1% penicillin-streptomycin (Gibco, Schwerte, Germany). The breast cancer cell lines and HEK293T were purchased from the Leibniz Institute DSMZ-German Collection of Microorganisms. All cells were cultured in a humidified atmosphere of 5% CO_2_ at 37 °C.

### 2.2. Anti-IgG-SNAP Nanobody Fusion Proteins Expression and Purification

The expression of the SNAP-tag fusion protein has been previously reported [25]. To generate anti-IgG-SNAP nanobody plasmids, the vector plasmid pMS-scFv-425-SNAP has been established previously [26], the insert nanobody plasmids pTP943, pTP1112, pTP1005, pTP1174 and pTP1183 were gifts from Dirk Görlich (Addgene plasmid # 104157, # 104158, # 104160, # 104162, # 104163, Table 1) [24]. The generated pMS-anti-IgG-SNAP nanobody plasmids were transfected into HEK293T cells with Roti^®^ Fect (Carl Roth, Karlsruhe, Germany). Successfully transfected cells were selected in culture medium containing 0.1 mg/mL Zeocin (InvivoGen, Toulouse, France). Since the anti-IgG-SNAP nanobody was cloned downstream of the immunoglobulin κ leader, the fusion proteins were released into culture medium. After collecting sufficient supernatant (500–600 mL), the proteins were purified using 10 mM, 40 mM and 250 mM imidazole in sequential steps with a Ni-NTA superflow cartridge (Qiagen, Hilden, Germany). All supernatant purifications were performed using an ÄKTA start system (GE Healthcare Bio-Sciences AB, Düsseldorf, Germany). The isolated fractions were incubated with SNAP-Surface^®^ Alexa Fluor^®^ 488 (New Englands Biolabs, Frankfurt am Main, Germany) for 20 min at room temperature and then loaded onto a 10% SDS-PAGE. The SDS-PAGE was imaged using a ChemiDoc XRS+ System (BIO-RAD, Hercules, CA, USA) to confirm the SNAP-tag’s properties. The presence of the anti-IgG-SNAP nanobody fusion protein was visualized by Coomassie brilliant blue staining.

### 2.3. Conjugation of Nbs Anti-IgG-SNAP Fusion Proteins with Benzylguanine-Modified Fluorescent Substrates

SNAP-Surface^®^ Alexa Fluor^®^ 488, SNAP-Surface^®^ Alexa Fluor^®^ 546, SNAP-Surface^®^ Alexa Fluor^®^ 594 and SNAP-Surface^®^ Alexa Fluor^®^ 647 (New Englands Biolabs) were conjugated to Nb anti-IgG-SNAP fusion proteins at a 2:1 ratio for 2 h at room temperature in the dark. Unbound reagents were removed using 7K MWCO Zeba ™ Spin Desalting Columns (Thermo Fisher Scientific, Schwerte, Germany). To confirm the efficiency of this site-specific conjugation, Alexa Fluor^®^ 647-labeled Nbs anti-IgG-SNAP were incubated with SNAP-Surface^®^ Alexa Fluor^®^ 488 and loaded onto an SDS-PAGE and imaged using the ChemiDoc XRS+ System and Odyssey DLx Imager (LI-COR Biosciences, Homburg, German) subsequently.

### 2.4. Fluorescence Western Blot

A total of 125 ng of primary antibodies were loaded onto a 10% SDS-PAGE without denaturation. These proteins were then transferred onto polyvinylidene difluoride (PVDF) membranes using the Trans-Blot^®^ Turbo™ transfer system (BIO-RAD). The membranes were blocked with western blot blocking buffer (1% BSA in TBST) for 1 h at room temperature, followed by incubation with Nb anti-IgG-SNAP-Surface^®^ Alexa Fluor^®^ 647 (1 µg/mL), Alexa Fluor™ Plus 647 conjugated with goat anti-mouse IgG (H+L) (Thermo Fisher Scientific, A32728, 1:8000) and Alexa Fluor™ 647 conjugated anti-rabbit IgG (H+L) (Thermo Fisher Scientific, A21245, 1:8000) for another 1 h individually. After incubation, the membranes were washed three times in TBST, followed by a final wash in TBS. The membranes were imaged using the Odyssey DLx Imager (LI-COR Biosciences).

### 2.5. Flow Cytometry

A total of 4 × 10^5^ cells of MDA-MB-468, MDA-MB-231, MDA-MB-453, Hs578T, MCF-7, 49Z, 12Z, Ishikawa and T-HESC cell lines were prepared. After washing twice with PBS, the cells were incubated with blocking buffer (1% BSA in PBS) on ice for 30 min. Primary antibodies (Table 2) were diluted in blocking buffer (1% BSA in PBS) on ice for 30 min. Following two washes with PBS, 1 µg of each Nb anti-IgG-SNAP-Surface^®^ Alexa Fluor^®^ 647 was diluted in 200 µL blocking buffer. Additionally, Alexa Fluor™ Plus 647 conjugated with goat anti-mouse IgG (H+L) (Thermo Fisher Scientific, A32728, 1:400) and Alexa Fluor™ 647 conjugated anti-rabbit IgG (H+L) (Thermo Fisher Scientific, A21245, 1:400) were incubated with cells on ice for 30 min separately. After two PBS washes, the cells were analyzed using a CytoFLEX Flow Cytometer (Beckman Coulter, Krefeld, Germany). For intracellular staining, cells were fixed in 4% formaldehyde and permeabilized with 0.1% Triton X-100. Blocking was performed using permeabilization buffer (10% FBS and 1% BSA in PBS). All antibodies and nanobodies were diluted in permeabilization buffer. The incubation steps and flow cytometry analysis were performed as described above. Data analysis was performed using the FlowJo software (Becton, 10.7.1 version, Ashland, OR, USA).

### 2.6. Fluorescence Microscopy

To visualize the binding of Nb anti-IgG-SNAP conjugated with SNAP Surface^®^ Alexa Fluor^®^ 647, the same cells used in flow cytometry were collected and seeded into a black 96-well microplate (Greiner Bio-One, Frickenhausen, Germany), at a density of 40,000 cells/well. The cells were incubated overnight at 37 °C. The following day, cells were fixed with 4% formaldehyde for 10 min and washed twice with PBS. Residual formaldehyde was quenched by applying 50 nM NH_4_Cl for 5 min. All subsequent steps followed the same protocol as described for flow cytometry. Antibodies and 1 µg Nb anti-IgG-SNAP-Surface^®^ Alexa Fluor^®^ 647 were diluted in 100 µL blocking buffer (1% BSA in PBS) for surface staining or in permeabilization buffer (0.3 (*v*/*v*) Triton X-100 in PBS) containing 10% FBS and 1% BSA for intracellular staining. Specific binding was visualized using a DMi8 S Live-cell microscope (Leica Microsystems, Wetzlar, Germany) equipped with a 100× oil objective.

### 2.7. Preparation of Cell Block

A total of 1 × 10^7^ endometrial cells (49Z, 12Z, Ishikawa and T-HESC) were collected and washed with PBS. Then, the cells were then fixed in 1 mL 4% formaldehyde overnight at room temperature. After fixation and subsequent washing with PBS, the cells were resuspended in 200 µL of 30 mg/mL Agarose Low Melt (Carl-Roth, Karlsruhe, Germany) in PBS at 42 °C. The solidified cell-agarose blocks were dehydrated through a graded series of ethanol (70%, 80%, 90%, 95%, 100%), and then kept in xylene overnight. Xylene was removed by incubating the blocks in liquid paraffin at 60 °C. Finally, the cell-agarose was embedded by melted paraffin.

### 2.8. Immunohistochemistry (IHC) of the Cell Blocks

Paraffin sections (4 µm) of the cell blocks were de-waxed and rehydrated through a graded series of ethanol (100%, 90%, 80%, 70%). After rinsing with distilled water, sections were microwaved in citrate buffer (10 mM trisodium citrate dihydrate, 0.05% Tween 20, pH 6.0) for 10 min for antigen retrieval. Following a PBS wash, the sections were immersed in 3% hydrogen peroxidase for 10 min to block endogenous peroxidase activity. Then the cell blocks were then blocked by 10% normal goat serum in PBS for 1 h at room temperature and incubated with the primary antibodies (Table 2) overnight at 4 °C. Afterward, the sections were staining with HRP conjugated anti-mouse IgG (Thermo Fisher Scientific, G21040, 1:100) or HRP conjugated anti-rabbit IgG (7074S, 1:500; Cell Signaling Technology, Leiden, The Netherlands). Visualization was performed using the Vector NovaRED Substrate Kit (Vector Laboratories, Newark, CA., USA), and the sections were counterstained with hematoxylin. The slides were imaged using the Axio Scan.Z1 Slide Scanner (Zeiss, Oberkochen, Germany) at 40× magnification. Semi-quantitative analysis was performed using the ImageJ IHC profiler (https://journals.plos.org/plosone/article?id=10.1371/journal.pone.0096801 (accessed on 19 February 2025)).

### 2.9. Multicolor Staining of the Cell Block

After de-waxing, rehydration, antigen retrieval and blocking, the cell blocks were incubated with primary antibodies (Table 3) overnight at 4 °C or 1 h at room temperature. Following three washes with PBST, the cell blocks were incubated for 1 h at room temperature with 2 µg of SNAP-Surface^®^ Alexa Fluor^®^ 488, SNAP-Surface^®^ Alexa Fluor^®^ 546, SNAP-Surface^®^ Alexa Fluor^®^ 594 or SNAP-Surface^®^ Alexa Fluor^®^ 647 (New Englands Biolabs) labeled Nb anti-IgG-SNAP according to the staining strategy and sequence showed in Table 3. After three additional washing steps with PBST and a final wash with PBS, the sections were mounted using Vectashield antifade mounting medium (H-1200, Vector Laboratories) containing 4′, 6-diamidino-2-phenylindole (DAPI). Finally, the cell blocks were imaged using a DMi8 S microscope (Leica Microsystems) equipped with a 100× oil objective and Instant Computational Clearing (ICC) method.

### 2.10. Statistical Analysis

The corrected total cell fluorescence (CTCF) data are presented as mean ± standard error of the mean (SEM). Statistical significance was determined using a one-way analysis of variance (ANOVA) followed by Dunnett’s multiple comparisons test, performed with GraphPad Prism 9.0.0. A *p*-value of * *p* < 0.05 was considered statistically significant.

## 3. Results

### 3.1. Production of Nb Anti-IgG-SNAP Fusion Protein

The Nb anti-IgG-SNAP fusion proteins (~39 kDa) were produced in transfected HEK293T cells and secreted into the cell culture supernatant. Since the fusion proteins contain the C-terminal 6× His-tag, they were enriched from the supernatant using an Ni-NTA superflow cartridge. The presence of fusion proteins and the self-labeling activity of SNAP-tag were confirmed by conjugating the collected fractions with the SNAP-Surface^®^ Alexa Fluor^®^ 488. SDS-PAGE visualized under UV-light by ChemiDoc XRS+ System revealed that while the fusion proteins were present at low concentrations in the cell culture supernatant (S), they were highly enriched in the fractions eluted with 250mM imidazole. As an example, Nb anti-mouse IgG1 Fc-SNAP is shown in Figure S1. Coomassie brilliant blue staining further confirmed that Nb anti-mouse IgG1 Fc-SNAP was only present in the fraction eluted with 250 mM imidazole. Following this purification protocol, other fusion proteins, including Nb anti-mouse IgG1 Fab-SNAP, Nb anti-mouse IgG2a Fc-SNAP, Nb anti-mouse kappa chain-SNAP and Nb anti-rabbit IgG Fc-SNAP were successfully enriched. This expression and purification method yielded up to 5 mg of protein per liter of cell culture medium.

### 3.2. Conjugation of Nb Anti-IgG-SNAP Fusion Proteins with Benzylguanine-Modified Fluorescent Substrates

**Figure 1:**
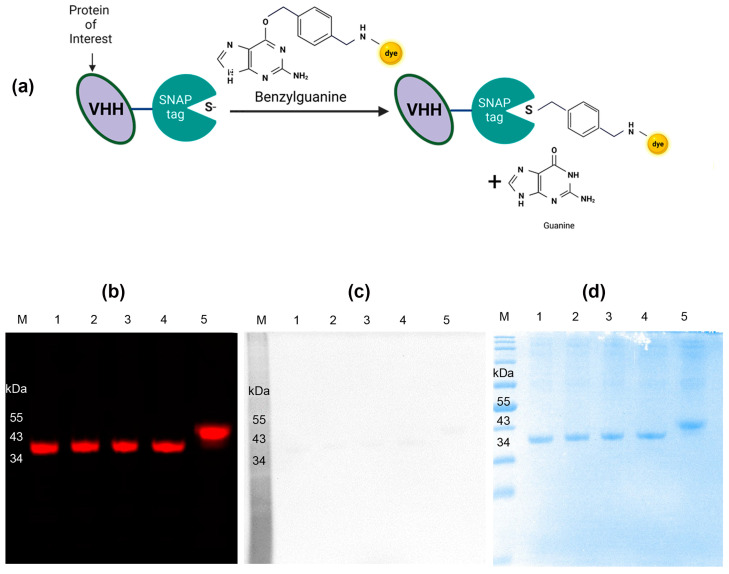
Nbs anti-IgG-SNAP conjugated with SNAP-Surface^®^ Alexa Fluor^®^ 647. (**a**) Mechanism of Nbs anti-IgG-SNAP conjugation. Created using BioRender (http://www.biorender.com). (**b**) Nbs anti-IgG-SNAP-647 were imaged by Odyssey DLx Imager. (**c**) Nbs anti-IgG-SNAP-647 were incubated with SNAP-Surface^®^ Alexa Fluor^®^ 488 and imaged by ChemiDoc XRS+ System. (**d**) Visualization of the corresponding SDS-PAGE in Coomassie brilliant blue staining. Lane 1: Nb anti-mouse IgG1 Fab-SNAP-647. Lane 2: Nb anti-mouse IgG1 Fc-SNAP-647. Lane 3: Nb anti-mouse IgG2a Fc-SNAP-647. Lane 4: Nb anti-mouse kappa chain-SNAP-647. Lane 5: Nb anti-rabbit IgG Fc-SNAP-647.

The conjugation of Nbs anti-IgG-SNAP with BG-modified fluorescence dyes was further investigated. Initially, Nbs anti-IgG-SNAP were conjugated with SNAP-Surface^®^ Alexa Fluor^®^ 647 (Figure 1a). To confirm the efficiency of SNAP-tag site-specific conjugation, Nbs anti-IgG-SNAP-647 were subsequently incubated with SNAP-Surface^®^ Alexa Fluor^®^ 488 and analyzed using the ChemiDoc XRS+ System. SDS-PAGE analysis followed by imaging of Alexa Fluor^®^ 647 signals with Odyssey DLx Imager exhibited a strong fluorescence signal (Figure 1b). In contrast, no Alexa Fluor^®^488 signal was detected when the same SDS-PAGE was visualized using the ChemiDoc XRS+ System (Figure 1c). The presence of all Nbs anti-IgG-SNAP fusion proteins in SDS-PAGE using Coomassie brilliant blue staining is shown in Figure 1d. These results confirmed that SNAP-tag was specifically and completely saturated with SNAP-Surface^®^ Alexa Fluor^®^ 647 after a 2 h incubation at room temperature with 2:1 molar ratio of BG-fluorescence dye to fusion protein. 

### 3.3. The Specific Binding of Nb Anti-IgG-SNAP Fusion Proteins in Fluorescence Western Blot

**Figure 2:**
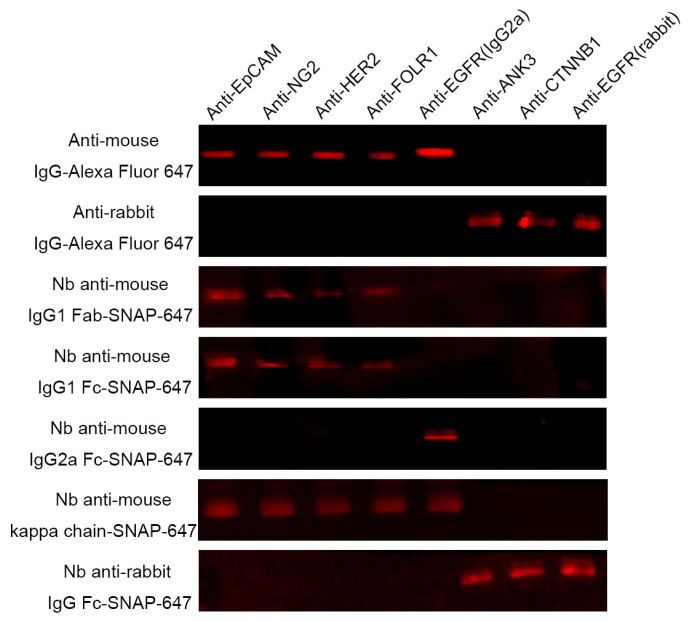
Binding-specificity analysis using fluorescence Western blot. Different primary antibodies representing different subtypes were loaded into the membrane and incubated with anti-mouse IgG-Alexa Fluor 647, anti-rabbit IgG-Alexa Fluor 647, Nb anti-mouse IgG1 Fab-SNAP-647, Nb anti-mouse IgG1 Fc-SNAP-647, Nb anti-mouse IgG2a Fc-SNAP-647, Nb anti-mouse kappa chain-SNAP-647 and Nb anti-rabbit IgG Fc-SNAP-647 separately. All the membranes were imaged under Odyssey DLx Imager. Subtypes of primary antibodies: anti-EpCAM (mouse/IgG1, kappa), anti-NG2 (mouse/IgG1, kappa), anti-HER2 (mouse/IgG1), anti-FOLR1 (mouse/IgG1), anti-EGFR (mouse/IgG2a), anti-ANK3 (rabbit/IgG), anti-CTNNB1 (rabbit/IgG), anti-EGFR (rabbit/IgG).

The ability of the fluorescence labeled Nbs anti-IgG-SNAP to specifically bind to primary antibodies with different subtypes was determined using fluorescence Western blot (Figure 2). Membranes containing different subtypes of mouse- or rabbit- derived primary antibodies were incubated with the purified Nbs anti-IgG-SNAP-Surface® Alexa Fluor® 647. Specific and strong signals were observed for mouse-derived primary antibodies, including anti-epithelial cell adhesion molecule (EpCAM) (IgG1, kappa), anti-nerve/glial-antigen 2 (NG2) (IgG1, kappa), anti-human epidermal growth factor receptor 2 (HER2) (IgG1) and anti-folate receptor alpha (FOLR1) (IgG1), when either Nb anti-mouse IgG1 Fab-SNAP-647 or Nb anti-mouse IgG1 Fc-SNAP-647 was used. Additionally, Nb anti-mouse IgG2a Fc-SNAP-647 reacted exclusively with mouse anti-EGFR IgG2a subtype antibody, anti-EGFR. Meanwhile, Nb anti-mouse kappa chain-SNAP-647 can recognize both mouse IgG1 subtype antibodies (anti-EpCAM, anti-NG2, anti-HER2 and anti-FOLR1) and mouse IgG2a subtype antibody (anti-EGFR). These results demonstrated that each Nb anti-mouse IgG-SNAP-647 exhibited unique and specific binding to the corresponding mouse monoclonal antibodies. For rabbit-derived primary antibodies, Nb anti-rabbit IgG Fc-SNAP-647 exhibited exclusive binding for anti-ANK3, anti-CTNNB1 and anti-EGFR (rabbit derived). As controls, commercially available secondary antibodies—anti-mouse IgG (H+L) and anti-rabbit IgG (H+L), both conjugated to Alexa Fluor 647—were also tested. Figure 2 demonstrated that Nbs anti-IgG-SNAP conjugated with Surface^®^ Alexa Fluor^®^ 647 exhibited specificity comparable to that of as commercial secondary antibodies and bind effectively to primary antibodies with consistent performance.

### 3.4. The Application of Nb Anti-IgG-SNAP Fusion Proteins as Secondary Antibodies for Flow Cytometry and Fluorescence Microscopy for Fixed Cells

**Figure 3:**
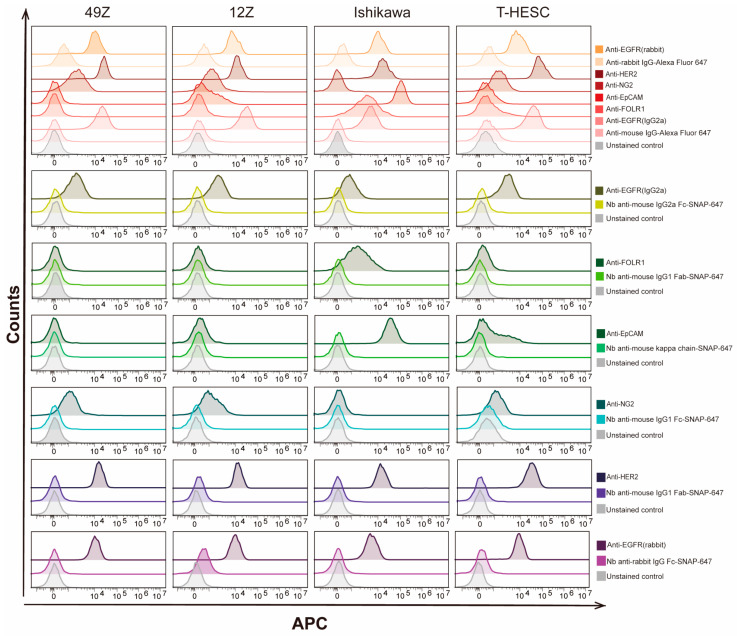
Flow cytometry analysis with Nbs anti-IgG-SNAP. Endometrial cells were incubated with anti-EGFR, anti-FOLR1, anti-EpCAM, anti-NG2 and anti-HER2, then they were detected with Surface^®^Alexa Fluor^®^ 647-labeled Nbs anti-IgG-SNAP or corresponding commercial antibodies.

**Figure 4:**
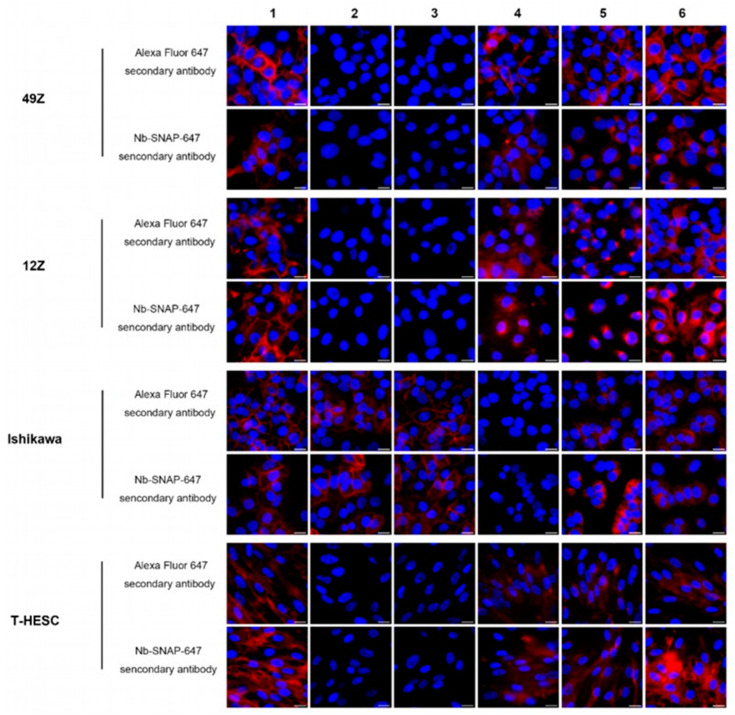
The specific binding of Nbs anti-IgG-SNAP was visualized by fluorescence microscopy. Primary antibodies targeting EGFR, FOLR1, EpCAM, NG2 and HER2 were incubated with endometrial cells followed by detecting with either commercial secondary antibodies conjugated with Alexa Fluor 647 or Nbs anti-IgG-SNAP- Alexa Fluor^®^ 647. The red signal presented Alexa Fluor^®^ 647 channel, blue signal was nuclear counterstain with Hoechst 33342. Lane1: anti-EGFR (IgG2a) antibody, detected by Nb anti-mouse IgG2a Fc-SNAP-647; Lane 2: anti-FOLR1 antibody, detected by Nb anti-mouse IgG1 Fab-SNAP-647; Lane 3: anti-EpCAM antibody, detected by Nb anti-mouse kappa chain-SNAP-647; Lane 4: anti-NG2 antibody, detected by Nb anti-mouse IgG1 Fc-SNAP-647; Lane 5: anti-HER2 antibody, detected by Nb anti-mouse IgG1 Fab-SNAP-647; Lane 6: anti-EGFR (rabbit) antibody, detected by anti-rabbit IgG Fc-SNAP-647 (scale bar 20 μm).

After confirming the specific activity of Nbs anti-IgG-SNAP-647 using fluorescence Western blot, their utility as secondary antibodies for IFA were explored and validated using flow cytometry and fluorescence microscopy. The expression levels of EpCAM, NG2, EGFR, FOLR1 and HER2 were determined in four different endometrial cell lines (49Z, 12Z, Ishikawa and T-HESC) and five breast cancer cell lines (MDA-MB-231, MDA-MB-468, Hs578T, MCF7 and MDA-MB-453) (Figure 3, Figure 4 and Figure S2), and summarized in Table 4 and Table S1. Using either commercial secondary antibodies or Nbs anti-IgG-SNAP-647 to detect different primary antibody subtypes, we observed that all endometrial cell lines expressed high levels of EGFR and HER2. FOLR1 and EpCAM were detected exclusively in Ishikawa cells, while NG2 was expressed in 49Z, 12Z and T-HESC. In breast cancer cell lines, the EGFR expression was highest in MDA-MB-468, moderate in MDA-MB-231 and Hs578T, low expressed in MCF7, and minimal in MDA-MB-453 (Figure S2e,f). MDA-MB-468, MCF7 and MDA-MB-453 expressed highest levels of EpCAM, while MDA-MB-231 and Hs578T showed negligible staining (Figure S2a,b). Strong binding signal was observed in Hs578T and medium signal in MDA-MB-231 when incubated with anti-NG2 antibody, and weak or no signal was detected in the other breast cancer cell lines (Figure S2c,d). All breast cell lines expressed ANK3 and CTNNB1 (Figure S2g–i). No significant differences were observed in the staining patterns when comparing commercial secondary antibodies to Nbs anti-IgG-SNAP-647 in both flow cytometry and fluorescence microscopy experiments (Figure 3, Figure 4 and Figure S2). Furthermore, these results confirmed that Nbs anti-IgG-SNAP can not only specifically recognize mouse- and rabbit-derived primary antibodies but also effectively distinguish between antibody subtypes produced within the same host.

### 3.5. Immunohistochemistry and Multicolor Immunofluorescence in Cell Blocks

**Figure 5:**
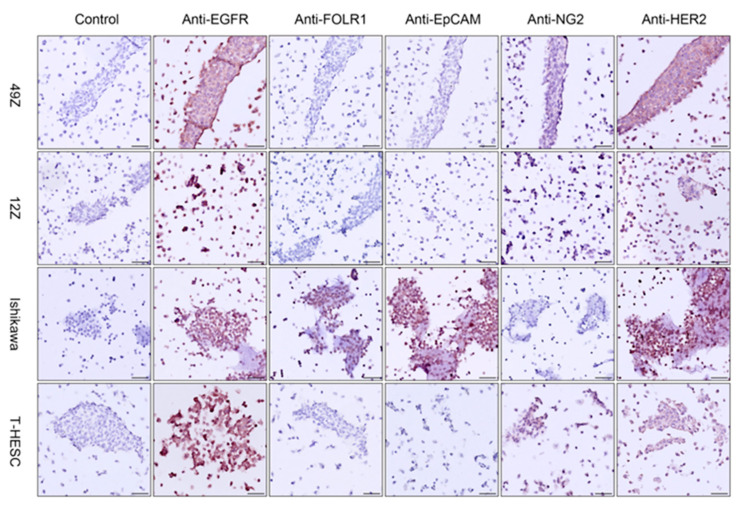
IHC images of endometriosis cell (49Z, 12Z, Ishikawa and T-HESC) blocks stained with anti-EGFR (detected by HRP conjugated anti-rabbit IgG), anti-FOLR1, anti-EpCAM, anti-NG2 and anti-HER2 (these five markers were detected by HRP conjugated anti-mouse IgG), separate-ly. EGFR and HER2 (brown color) were positive in all endometrial cells, EpCam and FOLR1 were only positive in Ishikawa cells. NG2 was positive in all cells except Ishikawa. Magnification is indicated by the black scale bars (50 μm).

Before performing multi-color immunofluorescence staining, IHC was used to confirm the expression levels of EGFR, FOLR1, EpCAM, NG2 and HER2 in cell blocks. All slides were scanned using Zeiss Axio Z1 scanner, and semi-quantitative image analysis was performed by the IHC profiler plug of software Image J, version 1.54k [27]. The IHC results aligned with the findings from flow cytometry and fixed-cell fluorescence microscopy, demonstrating high expression level of EGFR and HER2 across all endometrial cell lines. Notably, Ishikawa cells were negative for NG2, while EpCAM expression was exclusive in Ishikawa cells (Figure 5).

**Figure 6:**
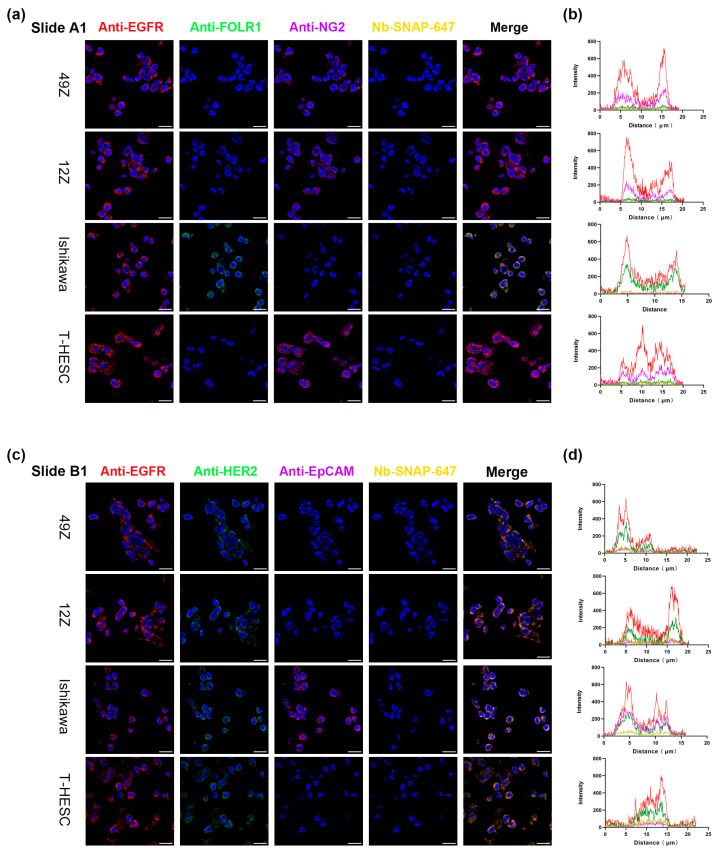
The full-setting multicolor staining of endometrial cell blocks. (**a**,**c**) Nbs anti-IgG-SNAP-488 (red), Nbs anti-IgG-SNAP-546 (green), Nbs anti-IgG-SNAP-594 (magenta) detecting different primary antibodies (anti-EGFR, anti-FOLR1, anti-NG2, anti-EpCAM, anti-HER2) or Nbs anti-IgG-SNAP-647 (yellow) post-incubation to confirming binding activity. (**b**,**d**) Fluorescence intensity profiles of SNAP-Surface^®^ Alexa Fluor^®^ 488 (red curve), SNAP-Surface^®^ Alexa Fluor^®^ 546 (green curve), SNAP-Surface^®^ Alexa Fluor^®^ 594 (magenta curve) and SNAP-Surface^®^ Alexa Fluor^®^ 647 (yellow curve). Magnification is indicated by the white scale bars (20 μm).

To explore the possibility of using Nbs anti-IgG-SNAP in multi-color immunofluorescence assay as secondary antibodies, we labeled Nbs anti-IgG-SNAP with different fluorescence dyes (SNAP-Surface^®^ Alexa Fluor^®^ 488, SNAP-Surface^®^ Alexa Fluor^®^ 546, SNAP-Surface^®^ Alexa Fluor^®^ 594, Surface^®^ Alexa Fluor^®^ 647) by following the previous conjugation protocol [25]. Subsequently, we designed a staining strategy that included Nbs anti-IgG-SNAP-647 to evaluate binding activity and incorporated leave-one-out control stains to assess the potential cross-reactivity among our Nbs anti-IgG-SNAP. This staining strategy included five target markers—EGFR, FOLR1, EpCAM, NG2 and HER2—whose expression had previously confirmed by IHC. For EGFR, we used two different primary antibodies derived from mouse and rabbit, respectively. Therefore, a total of six antibodies were used in this study. The experiments were conducted using two panels, Panel A and Panel B (Table 3). For example, in Panel A, A1 was firstly stained with anti-EGFR (IgG2a), detected by Nb anti-mouse IgG2a Fc-SNAP-488, followed by incubation with Nb anti-mouse IgG2a Fc-SNAP-647 to evaluate the binding activity. In the second staining round, A1 was incubated with anti-FOLR1 (IgG1), detected by Nb anti-mouse IgG1 Fab-SNAP-546, and subsequently post-incubated with Nb anti-mouse IgG1 Fab-SNAP-647. In the third round, A1 was stained with anti-NG2 (IgG1, kappa), then Nb anti-mouse IgG1 Fc-SNAP-594 and Nb anti-mouse IgG1 Fc-SNAP-647 were applied orderly (Figure 6a). Leave-one-out control stains were performed in A2, A3 and A4 (Figure S3a,c,e). Each row of images in Figure S3a,c,e represents staining with panel A, but with one of primary antibody omitted from the staining strategy. For example, A2 (Figure S3a) was stained with the same primary antibodies and Alexa Fluor dyes labeled Nbs anti-IgG-SNAP, but anti-EGFR (IgG2a) was excluded. Similarly, A3 (Figure S3c) omitted anti-FOLR1, and in A4 (Figure S3e) anti-NG2 was excluded. A control slide was also stained exclusively with Nbs anti-IgG-SNAP-Alexa Fluor dyes to measure the background signals (Figure S3g). The same protocol was applied in Panel B (Figure 6c and Figure S3b,d,f), which incubated with anti-EGFR (rabbit), anti-HER2 (IgG1) and anti-EpCAM (IgG1, kappa). These primary antibodies were detected with Nb anti-rabbit IgG Fc-SNAP-488, Nb anti-mouse IgG1 Fab-SNAP-546 and Nb anti-mouse kappa chain-SNAP-594, separately. Likewise, Alexa Fluor 647-labeled Nbs (Nb anti-rabbit IgG Fc-SNAP-647, Nb anti-mouse IgG1 Fab-SNAP-647, and Nb anti-mouse kappa chain-SNAP-647) were used to confirm the presence of binding activity. The control slide B was used to measure the background signals (Figure S3h). The multi-color immunofluorescence results were consistent with the IHC results and are summarized in Table 5. The findings showed high specificity and clear staining of targeted antigens, with no cross-reactivity observed among Alexa Fluor dyes labeled Nbs anti-IgG-SNAP.

**Figure 7:**
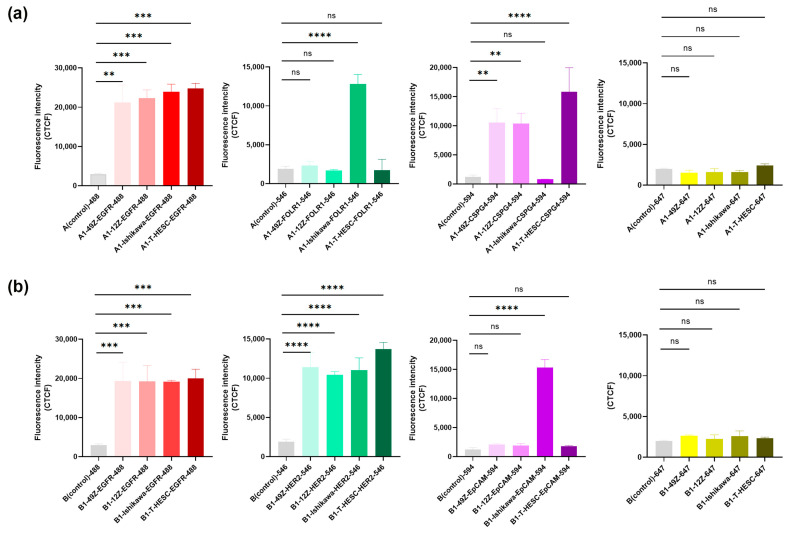
CTCF analysis of the fluorescence intensity of multicolor staining on cell block. (**a**) Comparing A1 slide with the A control slide in anti-EGFR (IgG2a)-Alexa Fluor 488 (red), anti-FOLR1-Alexa Fluor 546 (green), anti-NG2-Alexa Fluor 594 (magenta) and Nbs anti-IgG-SNAP-Alexa Fluor 647 (yellow). (**b**) Comparing B1 slide with the B control slide in anti-EGFR (rabbit)-Alexa Fluor 488 (red), anti-HER2-Alexa Fluor 546 (green), anti-EpCAM-Alexa Fluor 594 (magenta) and Nbs anti-IgG-SNAP-Alexa Fluor 647 (yellow). Data were analyzed by one-way analysis of variance (ANOVA) with Dunnett’s multiple comparisons test using GraphPad Prism 9.0.0 (ns, not significant, ** *p* <0.01, *** *p* <0.001, **** *p* <0.0001).

Next, the mean fluorescence intensity for each channel was measured using ImageJ software. For example, as shown in Table 5, Figure 6c, red signals corresponding to anti-EGFR (IgG2a)-488 and magenta signals corresponding to anti-NG2 (IgG1, kappa)-594 signals were detected. In contrast, no signals were observed for green-anti-FOLR1 (IgG1)-546 or yellow-Nbs anti IgG-SNAP-647 in 49Z. Similarly, Figure 6d demonstrates red signals from anti-EGFR (rabbit)-488 and green signals from anti-HER2 (IgG1)-546, while magenta-anti-EpCAM (IgG1, kappa)-594 and Nbs anti IgG-SNAP-647 showed no detectable signals in 49Z. The fluorescent intensity profiles of leave-one-out controls are shown in Figure S3, confirming no cross-reactivity between the Nbs anti-IgG-SNAP in multicolor staining. Since no cross-reactivity was observed, fluorescence intensity analysis focused on comparing A (control) with A1, and B (control) with B1 (Figure 7), using the CTCF with ImgaJ software. For Alexa Fluor 488 channel (red), both anti-EGFR (IgG2a) and anti-EGFR (rabbit) showed a significant difference. In the Alexa Fluor 546 channel (green), only Ishikawa cells exhibited significant differences with anti-FOLR1 (IgG1), while all endometrial cells showed a high signal intensity with anti-HER2 (IgG1). For Alexa Fluor 594 channel (magenta), Ishikawa cells displayed significant differences with anti-EpCAM (IgG1, kappa), while 49Z, 12Z and T-HESC cells exhibited distinguishable differences with anti-NG2 (IgG1, kappa). However, in the Alexa Fluor 647 channel (yellow), no significant differences were observed between A1/B1 and their respective controls (A control/B control). The CTCF analysis results were consistent with the data obtained from flow cytometry, fluorescence microscopy and immunohistochemistry.

## 4. Discussion

IFA is a technology that uses fluorescent-labeled antibodies to detect target mole-cules. While direct immunofluorescence assay offers a shorter protocol and reduced background noise, IFA remains widely favorable due to its high sensitivity, flexibility in selecting fluorescent colors, strong signal intensity and cost-effectiveness. As a result, IFA is more commonly used than the direct method, and there is a high global demand for fluorophore-labeled secondary antibodies. The European Union’s Directive 2010/63/EU addresses concern regarding the ethical use of animals in scientific re-search by emphasizing the 3Rs—Replacement, Reduction, and Refinement—which aim to minimize animal sacrifice in research [28]. Additionally, batch-to-batch variability in secondary antibodies remains a significant challenge, often affecting reproducibility. Conventional secondary antibody production is also time-consuming and expensive, further underscoring the need for alternative approaches. To address these limitations, technological innovations have introduced in vitro methods for recombinant antibody production. These methods provide consistent and reliable alternatives to traditionally animal-produced antibodies, improving standardization and reproducibility in research applications [29]. Currently, commercial secondary antibodies are typically produced in animals and later conjugated to fluorescent dyes for IFA. In this work, we aim to innovate secondary antibody production by employing SNAP-tag technology [25] in mammalian cell expression systems. We also incorporate anti-mouse and anti-rabbit IgG secondary nanobodies [24], providing promising alternatives to conventional secondary antibodies in IFA.

Nbs have been successfully expressed in various expression systems, including bacterial, yeast, plants and mammalian cells [30,31]. However, non-mammalian systems are often associated with limitations such as low yield, occasional N-glycosylation, and transgene instability [32,33]. In contrast, mammalian cell expression systems are considered the most favorable method for producing functional antibodies [34], particularly due to their ability to perform human-like post-translational modifications (PTMs), which are crucial for therapeutic antibodies [35]. These systems can produce up to 3–8 g/L of recombinant antibodies in culture [36]. Moreover, mammalian cell expression offers consistent and stable genetic engineering, enabling the production of antibodies with precise and well-defined characteristics, thereby improving reproducibility. Importantly, this in vitro technology minimizes or eliminates animal use while supporting rapid and large-scale production, making it an ideal platform for the characterization of human-associated proteins [37]. For this study, the HEK293T expression system was chosen to produce Nbs anti-IgG-SNAP due to its highly efficient transfection capacities, rapid growth, adaptability to diverse culture conditions, capacity for PTMs, and ability to produce a wide range of proteins [38]. Using this system, Nb anti-IgG-SNAP fusion proteins were produced at yields of up to 5 mg/L from 500 mL of supernatant. As shown in Figure 1c, the size of Nbs anti-IgG-SNAP is approximately 39 kDa, which is significantly smaller than commercial secondary antibodies (~150 kDa). This smaller size facilitates deeper and more even penetration into tissues, improving the detection of target molecules in complex tissues. Furthermore, as depicted in Figure 1a, Nbs consist of a single antigen-binding domain, enabling precise epitope targeting while reducing non-specific binding and background signals [39].

Next, the fluorescence labeling strategy was considered. To date, N-hydroxysuccinimide (NHS) ester, cysteine-maleimide and sortase A have been the most commonly used methods for labeling Nbs with fluorophores in immunofluorescence assays [24,40,41]. However, compared to the SNAP-tag technology, the following occurred: 1) NHS ester randomly labels lysine residues, resulting in nanobodies are heterogeneous [42]; 2) cysteine-maleimide labeling often results in low yields, dimerization and risks of irreversible unfolding [43], and this labeling reaction generally allows only single-site binding while requiring modification of POIs existing native Cys [44]; 3) the sortase A-reaction is reversible and requires large excesses of substrates to achieve a labeling reaction with low efficiency [45], with another drawback being the limited availability of modified fluorophores. In contrast, SNAP-tag, a self-labeling protein, offers several advantages. SNAP-tag is relatively small and can be positioned at the N-terminus or C-terminus of POIs without impairing their functionality [10,11]. Moreover, SNAP-tag enables superior stoichiometry, higher site specificity, and faster reactions by specifically conjugating to BG-modified fluorophores [46]. We have previously developed a one-step site-specific SNAP-tag conjugation strategy for single-chain antibody fragment (scFv) using BG-modified molecules [25]. This approach provides an efficient and reliable alternative for fluorophore labeling, overcoming the challenges associated with conventional methods. On the other hand, a variety of BG-fluorophores are currently available, offering researchers a wide range of options for divers staining strategies. Using our straightforward conjugation protocol, SNAP-Surface^®^ Alexa Fluor^®^ 647 was conjugated to Nbs anti-IgG-SNAP following purification. This protocol involves incubating Nbs anti-IgG-SNAP with BG-fluorophores in PBS buffer for 2 h in the dark, resulting in the generation of Nbs anti-IgG-SNAP-Surface^®^ Alexa Fluor^®^ 647 as irreversible labeling fluorescent probes. Figure 1b and Figure 1c demonstrate that using 2:1 molar ratio of SNAP-Surface^®^ Alexa Fluor^®^ 647 to Nbs anti-IgG-SNAP effectively blocks all active binding-sites of SNAP-tag, confirming the complete labeling. The Nbs anti-IgG-SNAP-647 conjugates were stored at −20 ℃ in PBS buffer and remained functional for at least 2–4 weeks without noticeable loss of performance, and further investigation into long-term stability and storage conditions will be considered in future work. Additionally, in this study, Figure 2 illustrates the specificity of Nbs anti-IgG-SNAP-647 for different subclasses of mouse- and rabbit-produced primary antibodies, which was the primary goal of the fluorescence Western blot. Notably, Nb anti-mouse kappa chain-SNAP reacted with all mice monoclonal antibodies tested, reflecting the predominance of kappa light chains in mouse IgGs. This finding aligns with established knowledge that kappa chains are overwhelmingly prevalent in mouse monoclonal Abs (kappa: lambda = 99:1). The results demonstrated that Nbs anti-IgG-SNAP-Alexa Fluor^®^ 647 exhibited species specificity comparable to commercial Alexa Fluor^®^ 647 conjugated secondary antibodies, effectively distinguishing mouse- and rabbit-produced primary antibodies. Specifically, the four Nbs anti-mouse IgG-SNAP-647 bound exclusively to mouse-produced antibodies, while Nb anti-rabbit IgG Fc-SNAP-647 selectively recognized rabbit-derived antibodies, consistent with a previous study [24].

In this study, various breast cancer cell lines and endometrial cells were used to analyze different expression levels of multiple target antigens (EGFR, FOLR1, HER2, EpCAM, NG2, ANK3 and CTNNB1) through flow cytometry and fluorescence microscopy (Figure 3, Figure 4 and Figure S2, Table 4 and Table S1). The Nbs anti-IgG-SNAP-647 produced results consistent with previous studies [26,47,48,49,50,51,52,53,54,55,56,57,58,59,60]. When comparing the fluorescence intensities of Nb anti-mouse IgG2a Fc-SNAP-647 and Nb anti-rabbit IgG Fc-SNAP-647 with commercial antibodies in flow cytometry, their signals appeared relatively weaker. This phenomenon is likely due to differences in the labeling strategies. Commercial secondary antibodies labeled with Alexa Fluor 647 (Thermo Fisher Scientific) generally incorporate 2–8 fluorophores per IgG molecule, leading to significant signal enhancement, particularly when detecting targets present in low quantities. In contrast, Nbs anti-IgG-SNAP utilizes a 1:1 binding ratio, where a single SNAP-Surface^®^ Alexa Fluor^®^ 647 conjugates with a Nbs anti-IgG-SNAP [9,61]. Despite this, Nbs anti-IgG-SNAP-Alexa Fluor^®^ 647 not only exhibited fluorescence intensities and quantities comparable to those of commercial Alexa Fluor 647 conjugated secondary antibodies but also accurately reflected low or high expression levels of target proteins in breast cancer cell lines and endometrial cells. This demonstrates their effectiveness in detecting primary antibodies while preserving target specificity. Additionally, Nbs anti-IgG-SNAP-Alexa Fluor^®^ 647 generated high-resolution signals comparable to those of commercial secondary antibodies in fluorescence microscopy. Furthermore, their smaller size (~39 kDa) enhanced the precision of protein localizations, providing a distinct advantage over larger commercial IgGs [62].

After evaluating the performance of flow cytometry and fluorescence microscopy, we explored the possibility of multi-color immunofluorescence staining in cell blocks, given that SNAP-tag technology has been reported for multicolor imaging in live-cell through its reaction with various BG-fluorophore substrates. A cell block is a paraffin-embedded technique utilized for immunohistochemistry and long-term storage, serving as a critical bridge between cytology and histology in diagnostic applications [63]. Based on these considerations, we prepared cell blocks for immunohistochemistry and multicolor immunofluorescence. Figure 5 and Figure 6 present results consistent with those obtained from flow cytometry and fluorescence microscopy in endometrial cell blocks. The leave-one-out staining controls strategy (Table 3) and post-staining with Nbs anti-IgG-SNAP-Alexa Fluor^®^ 647 demonstrated no cross-reactivity between previously applied Nbs anti-IgG-SNAP-Alexa Fluor^®^ dyes and subsequently applied Nbs anti-IgG-SNAP-Alexa Fluor^®^ dyes (Figure 6 and Figure S3), confirming the specificity of Nbs anti-IgG-SNAP-Alexa Fluor^®^ dyes as secondary antibodies. Previous studies have reported that recombinant nanobody proteins were expressed in HEK293T cells and then conjugated to Alexa Fluor 488, ATTO 488, or ATTO 647 using the NHS ester method for immunofluorescence experiments [64,65]. Additionally, SNAP-tag technology has been combined with CLIP-tag or Halo tag technology to develop a dual-labeling, allowing the detection of two target molecules [66,67,68]. However, these methods function similarly to primary antibodies in direct immunofluorescence assays, requiring designing and expressing different construct, thereby necessitating the generating of numerous recombinant antibodies for various target antigens. Moreover, unlike pre-conjugated commercial secondary antibodies—which are limited to fixed fluorophores, costly, involving complex protocols, and may result in spectral overlap in multicolor immunofluorescence—engineered Nbs anti-IgG-SNAP provided precise tagging without spectral interference, making them highly suitable for multicolor imaging. Additionally, due to the SNAP-tag, the Nbs anti-IgG-SNAP allow for flexible and customized fluorophore labeling, enhancing multiplexing and spectral compatibility. Overall, our work has been successful in developing fluorescence-labeled recombinant antibodies as secondary antibodies in IFA, based on the self-labeling SNAP-tag and previously established anti-mouse and anti-rabbit IgG nanobodies, which were expressed in mammalian cell system. By following a simple conjugation protocol for the SNAP-tag with BG-modified fluorophores, all fluorescence-labeled Nbs anti-IgG-SNAP demonstrated high specificity for primary antibodies in fluorescent Western blot, flow cytometry, fluorescence microscopy and multicolor immunofluorescence, yielding results comparable to those of commercial secondary antibody.

## 5. Conclusions

This study demonstrates the innovative recombinant secondary antibody method offers a novel strategy for antibody production that reduces animal usage and is cost-effective. Additionally, the fluorescence labeling reaction occurs under simple conditions, further enhancing the advantage of Nbs anti-IgG-SNAP. These features make them a competitive alternative to traditional secondary antibodies in IFA and positioning them as potential fluorescent probes for diagnosis and imaging applications.

## Figures and Tables

**Table 1:** List of nanobody encoding plasmids.

Plasmid name	Catalog no.	Features
pTP943	104157	Anti-mouse IgG1 Fab specific nanobody TP886
pTP1112	104158	Anti-mouse IgG1 Fc specific nanobody TP1107
pTP1005	104160	Anti-mouse IgG2a Fc specific nanobody TP1129
pTP1174	104162	Anti-mouse kappa (κ) chain specific nanobody TP1170
pTP1183	104163	Anti-rabbit IgG Fc specific nanobody TP897

**Table 2:** List of primary antibodies used for staining.

Antibody	Class and Isotype	Dilution	Supplier	Catalog-No
Anti-EGFR	Monoclonal mouse/IgG2a	IF: 1:200 Multi-IF: 1:20	Invitrogen, Schwerte, Germany	MA5-13319
Anti-EGFR	Recombinant monoclonal rabbit/IgG	IF: 1:100	Invitrogen, Schwerte, Germany	700308
Anti-EGFR	Polyclonal rabbit/IgG	IHC: 1:200 Multi-IF: 1:200	Sigma-Aldrich, Taufkirchen, Germany	HPA018530
Anti-EpCAM	Monoclonal mouse/IgG1, kappa	IF: 1:200 IHC: 1:50 Multi-IF: 1:50	Invitrogen, Schwerte, Germany	14-9326-82
Anti-NG2	Monoclonal mouse/IgG1, kappa	IF: 1:100 IHC: 1:100 Multi-IF: 1:100	Invitrogen, Schwerte, Germany	37-2700
Anti-HER2	Monoclonal Mouse/IgG1	IF: 1:100 IHC: 1:500 Multi-IF: 1:500	Invitrogen, Schwerte, Germany	MA5-13675
Anti-FOLR1	Monoclonal mouse/IgG1	IF: 1:100 IHC: 1:50 Multi-IF: 1:50	Invitrogen, Schwerte, Germany	MA5-23917
Anti-CTNNB1	Polyclonal rabbit/IgG	IF: 1:200	Sigma-Aldrich, Taufkirchen, Germany	HPA029159
Anti-ANK3	Polyclonal rabbit/IgG	IF: 1:100	Sigma-Aldrich, Taufkirchen, Germany	HPA038455

IF: immunofluorescence; IHC: immunohistochemistry; EGFR: epidermal growth factor receptor; HER2: human epidermal growth factor receptor 2; EpCAM: epithelial cell adhesion molecule; NG2: nerve/glial-antigen 2; FOLR1: folate receptor alpha; CTNNB1: beta Catenin 1; ANK3: ankyrin 3.

**Table 3:** The strategy and sequence of multicolor staining.

		Panel A						Panel B				
Staining Sequence	Antibody	A1	A2	A3	A4	A Control	Antibody	B1	B2	B3	B4	B Control
1	Anti-EGFR (mouse)	+ ^1^	− ^2^	+	+	−	Anti-EGFR (rabbit)	+	−	+	+	−
2	Nb anti-mouse IgG2a Fc-SNAP-488	+	+	+	+	+	Nb anti-rabbit IgG Fc-488	+	+	+	+	+
3	Nb anti-mouse IgG2a Fc-SNAP-647	+	+	+	+	+	Nb anti-rabbit IgG Fc-647	+	+	+	+	+
4	Anti-FOLR1	+	+	−	+	−	Anti-HER2	+	+	−	+	−
5	Nb anti-mouse IgG1 Fab-SNAP-546	+	+	+	+	+	Nb anti-mouse IgG1 Fab-SNAP-546	+	+	+	+	+
6	Nb anti-mouse IgG1 Fab-SNAP-647	+	+	+	+	+	Nb anti-mouse IgG1 Fab-SNAP-647	+	+	+	+	+
7	Anti-NG2	+	+	+	−	−	Anti-EpCAM	+	+	+	−	−
8	Nb anti-mouse IgG1 Fc-SNAP-594	+	+	+	+	+	Nb anti-mouse kappa chain-SNAP-594	+	+	+	+	+
9	Nb anti-mouse IgG1 Fc-SNAP-647	+	+	+	+	+	Nb anti-mouse kappa chain-SNAP-647	+	+	+	+	+

^1^ “+” presents add this antibody; ^2^ “−” presents not add this antibody.

**Table 4:** Different antigens expression levels in endometrial cell lines.

Target	49Z	12Z	Ishikawa	T-HESC
EGFR	high	high	high	high
HER2	high	high	high	high
NG2	medium	medium	low	medium
EpCAM	low	low	high	low
FOLR1	low	low	high	low

**Table 5:** Summary of multi-color fluorescence staining results.

	Panel A					Panel B				
Endometrial cells	Slide	488 anti-EGFR	546 anti-FOLR1	594 anti-NG2	647 channel	Slide	488 anti-EGFR	546 anti-HER2	594 anti-EpCAM	647 channel
49Z	A1	+ ^1^	− ^2^	+	−	B1	+	+	−	−
	A2	−	−	+	−	B2	−	+	−	−
	A3	+	−	+	−	B3	+	−	−	−
	A4	+	−	−	−	B4	+	+	−	−
	A control	−	−	−	−	B control	−	−	−	−
12Z	A1	+	−	+	−	B1	+	+	−	−
	A2	-	−	+	−	B2	−	+	−	−
	A3	+	−	+	−	B3	+	−	−	−
	A4	+	−	−	−	B4	+	+	−	−
	A control	-	−	−	−	B control	−	−	−	−
Ishikawa	A1	+	+	−	−	B1	+	+	+	−
	A2	−	+	−	−	B2	−	+	+	−
	A3	+	−	−	−	B3	+	−	+	−
	A4	+	+	−	−	B4	+	+	−	−
	A control	−	−	−	−	B control	−	−	−	−
T-HESC	A1	+	−	+	−	B1	+	+	−	−
	A2	−	−	+	−	B2	−	+	−	−
	A3	+	−	+	−	B3	+	−	−	−
	A4	+	−	−	−	B4	+	+	−	−
	A control	−	−	−	−	B control	−	−	−	−

^1^ “+” presents positive staining. ^2^ “−” presents negative staining.

## Data Availability

The original contributions presented in this study are included in the article/supplementary material. Further inquiries can be directed to the corresponding author.

## Supplementary Materials

The following supporting information can be downloaded at: https://www.mdpi.com/article/10.3390/cells14100691/s1, Figure S1: Enrichment of Nb anti-IgG1 Fc-SNAP; Figure S2: Fluorescence microscope and flow cytometry analysis with Nb anti-IgG-SNAP in breast cancer cell lines. Figure S3: The multicolor immunofluorescence and fluorescence intensity profiles of the leave-one-out control strategy and Nbs anti-IgG-SNAP-647 post-incubation; Table S1: Different antigens expression levels in breast cancer cell lines.

## Abbreviations

The following abbreviations are used in this manuscript:

FPs	Fluorescent proteins
GFP	Green fluorescent protein
POI	Protein of interest
hAGT	O^6^-alkylguanine-DNA alkyltransferase
BG	Benzylguanine
Nb	Nanobody
VHH	Variable heavy chain
CDRs	Complementarity determining regions
IFA	Indirect immunofluorescence assay
FBS	Fetal calf serum
ITS-X	Insulin-transferrin-selenium-ethanolamine
PVDF	Polyvinylidene difluoride
IHC	Immunohistochemistry
ICC	Instant computational clearing
CTCF	Corrected total cell fluorescence
PTMs	Post-translational modifications
NHS	N-hydroxysuccinimide
scFv	Single-chain antibody fragment
EGFR	Epidermal growth factor receptor
EpCAM	Epithelial cell adhesion molecule
NG2	Nerve/glial-antigen 2
FOLR1	Folate receptor alpha
CTNNB1	beta Catenin 1
ANK3	ankyrin 3

## References

[1] Tanenbaum M.E., Gilbert L.A., Qi L.S., Weissman J.S., Vale R.D. (2014). A protein-tagging system for signal amplification in gene expression and fluorescence imaging. Cell.

[2] Hoffman R.M. (2005). The multiple uses of fluorescent proteins to visualize cancer in vivo. Nat. Rev. Cancer.

[3] Giepmans B.N., Adams S.R., Ellisman M.H., Tsien R.Y. (2006). The fluorescent toolbox for assessing protein location and function. Science.

[4] Tsien R.Y. (1998). The green fluorescent protein. Annu. Rev. Biochem..

[5] Chalfie M., Tu Y., Euskirchen G., Ward W.W., Prasher D.C. (1994). Green fluorescent protein as a marker for gene expression. Science.

[6] Shaner N.C., Steinbach P.A., Tsien R.Y. (2005). A guide to choosing fluorescent proteins. Nat. Methods.

[7] Xia T., Li N., Fang X. (2013). Single-molecule fluorescence imaging in living cells. Annu. Rev. Phys. Chem..

[8] Pegg A.E. (2000). Repair of O6-alkylguanine by alkyltransferases. Mutat. Res. /Rev. Mutat. Res..

[9] Keppler A., Kindermann M., Gendreizig S., Pick H., Vogel H., Johnsson K. (2004). Labeling of fusion proteins of O6-alkylguanine-DNA alkyltransferase with small molecules in vivo and in vitro. Methods.

[10] Keppler A., Pick H., Arrivoli C., Vogel H., Johnsson K. (2004). Labeling of fusion proteins with synthetic fluorophores in live cells. Proc. Natl. Acad. Sci. USA.

[11] Srikun D., Albers A.E., Nam C.I., Iavarone A.T., Chang C.J. (2010). Organelle-targetable fluorescent probes for imaging hydrogen peroxide in living cells via SNAP-Tag protein labeling. J. Am. Chem. Soc..

[12] Cole N.B. (2013). Site-specific protein labeling with SNAP-tags. Curr. Protoc. Protein Sci..

[13] Kampmeier F., Ribbert M., Nachreiner T., Dembski S., Beaufils F., Brecht A., Barth S.J.B.c. (2009). Site-specific, covalent labeling of recombinant antibody fragments via fusion to an engineered version of 6-O-alkylguanine DNA alkyltransferase. Bioconjugate Chem..

[14] Cortez-Retamozo V., Backmann N., Senter P.D., Wernery U., De Baetselier P., Muyldermans S., Revets H. (2004). Efficient cancer therapy with a nanobody-based conjugate. Cancer Res..

[15] Decanniere K., Desmyter A., Lauwereys M., Ghahroudi M.A., Muyldermans S., Wyns L. (1999). A single-domain antibody fragment in complex with RNase A: Non-canonical loop structures and nanomolar affinity using two CDR loops. Structure.

[16] Wesolowski J., Alzogaray V., Reyelt J., Unger M., Juarez K., Urrutia M., Cauerhff A., Danquah W., Rissiek B., Scheuplein F. (2009). Single domain antibodies: Promising experimental and therapeutic tools in infection and immunity. Med. Microbiol. Immunol..

[17] De Genst E., Silence K., Decanniere K., Conrath K., Loris R., Kinne J., Muyldermans S., Wyns L. (2006). Molecular basis for the preferential cleft recognition by dromedary heavy-chain antibodies. Proc. Natl. Acad. Sci. USA.

[18] Decanniere K., Muyldermans S., Wyns L. (2000). Canonical antigen-binding loop structures in immunoglobulins: More structures, more canonical classes?. J. Mol. Biol..

[19] Herce H.D., Deng W., Helma J., Leonhardt H., Cardoso M.C. (2013). Visualization and targeted disruption of protein interactions in living cells. Nat. Commun..

[20] Mujic-Delic A., de Wit R.H., Verkaar F., Smit M.J. (2014). GPCR-targeting nanobodies: Attractive research tools, diagnostics, and therapeutics. Trends Pharmacol. Sci..

[21] Gray A., Bradbury A.R.M., Knappik A., Pluckthun A., Borrebaeck C.A.K., Dubel S. (2020). Animal-free alternatives and the antibody iceberg. Nat. Biotechnol..

[22] Gorovits B., Hays A., Jani D., Jones C., King C., Lundequist A., Mora J., Partridge M., Pathania D., Ramaswamy S.S. (2021). AAPS Perspective on the EURL Recommendation on the use of Non-Animal-Derived Antibodies. AAPS J..

[23] Shen H.J.N.N. (2013). Discovery of goat facility adds to antibody provider’s woes. Nature.

[24] Pleiner T., Bates M., Gorlich D. (2018). A toolbox of anti-mouse and anti-rabbit IgG secondary nanobodies. J. Cell Biol..

[25] Hussain A.F., Heppenstall P.A., Kampmeier F., Meinhold-Heerlein I., Barth S. (2019). One-step site-specific antibody fragment auto-conjugation using SNAP-tag technology. Nat. Protoc..

[26] Zhang C., Sheng W., Al-Rawe M., Mohiuddin T.M., Niebert M., Zeppernick F., Meihold-Heerlein I., Hussain A.F. (2022). EpCAM- and EGFR-Specific Antibody Drug Conjugates for Triple-Negative Breast Cancer Treatment. Int. J. Mol. Sci..

[27] Varghese F., Bukhari A.B., Malhotra R., De A. (2014). IHC Profiler: An open source plugin for the quantitative evaluation and automated scoring of immunohistochemistry images of human tissue samples. PLoS ONE.

[28] EC (2010). Directive 2010/63/EU of the European Parliament and of the Council of 22 September 2010 on the protection of animals used for scientific purposes. Off. J. Eur. Union.

[29] Kunert R., Reinhart D. (2016). Advances in recombinant antibody manufacturing. Appl. Microbiol. Biotechnol..

[30] Liu Y., Huang H. (2018). Expression of single-domain antibody in different systems. Appl. Microbiol. Biotechnol..

[31] Muyldermans S. (2001). Single domain camel antibodies: Current status. J. Biotechnol..

[32] Virdi V., Coddens A., De Buck S., Millet S., Goddeeris B.M., Cox E., De Greve H., Depicker A. (2013). Orally fed seeds producing designer IgAs protect weaned piglets against enterotoxigenic Escherichia coli infection. Proc. Natl. Acad. Sci. USA.

[33] Lentz E.M., Garaicoechea L., Alfano E.F., Parreno V., Wigdorovitz A., Bravo-Almonacid F.F. (2012). Translational fusion and redirection to thylakoid lumen as strategies to improve the accumulation of a camelid antibody fragment in transplastomic tobacco. Planta.

[34] Kooijmans S.A.A., Gitz-Francois J., Schiffelers R.M., Vader P. (2018). Recombinant phosphatidylserine-binding nanobodies for targeting of extracellular vesicles to tumor cells: A plug-and-play approach. Nanoscale.

[35] Dhara V.G., Naik H.M., Majewska N.I., Betenbaugh M.J. (2018). Recombinant Antibody Production in CHO and NS0 Cells: Differences and Similarities. BioDrugs.

[36] Kelley B., Kiss R., Laird M. (2018). A Different Perspective: How Much Innovation Is Really Needed for Monoclonal Antibody Production Using Mammalian Cell Technology?. Adv. Biochem. Eng. Biotechnol..

[37] Aricescu A.R., Lu W., Jones E.Y. (2006). A time- and cost-efficient system for high-level protein production in mammalian cells. Acta Crystallogr. D Biol. Crystallogr..

[38] Tan E., Chin C.S.H., Lim Z.F.S., Ng S.K. (2021). HEK293 Cell Line as a Platform to Produce Recombinant Proteins and Viral Vectors. Front. Bioeng. Biotechnol..

[39] Alexander E., Leong K.W. (2024). Discovery of nanobodies: A comprehensive review of their applications and potential over the past five years. J. Nanobiotechnol..

[40] Fridy P.C., Li Y., Keegan S., Thompson M.K., Nudelman I., Scheid J.F., Oeffinger M., Nussenzweig M.C., Fenyö D., Chait B.T. (2014). A robust pipeline for rapid production of versatile nanobody repertoires. Nature Methods.

[41] Massa S., Vikani N., Betti C., Ballet S., Vanderhaegen S., Steyaert J., Descamps B., Vanhove C., Bunschoten A., van Leeuwen F.W. (2016). Sortase A-mediated site-specific labeling of camelid single-domain antibody-fragments: A versatile strategy for multiple molecular imaging modalities. Contrast Media Mol. Imaging.

[42] Schumacher D., Helma J., Schneider A.F.L., Leonhardt H., Hackenberger C.P.R. (2018). Nanobodies: Chemical Functionalization Strategies and Intracellular Applications. Angew. Chem. Int. Ed. Engl..

[43] Pleiner T., Bates M., Trakhanov S., Lee C.T., Schliep J.E., Chug H., Bohning M., Stark H., Urlaub H., Gorlich D. (2016). Correction: Nanobodies: Site-specific labeling for super-resolution imaging, rapid epitope-mapping and native protein complex isolation. Elife.

[44] Fung H.Y.J., McKibben K.M., Ramirez J., Gupta K., Rhoades E. (2020). Structural Characterization of Tau in Fuzzy Tau:Tubulin Complexes. Structure.

[45] Morgan H.E., Turnbull W.B., Webb M.E. (2022). Challenges in the use of sortase and other peptide ligases for site-specific protein modification. Chem. Soc. Rev..

[46] Wilhelm J., Kuhn S., Tarnawski M., Gotthard G., Tunnermann J., Tanzer T., Karpenko J., Mertes N., Xue L., Uhrig U. (2021). Kinetic and Structural Characterization of the Self-Labeling Protein Tags HaloTag7, SNAP-tag, and CLIP-tag. Biochemistry.

[47] Gostner J.M., Fong D., Wrulich O.A., Lehne F., Zitt M., Hermann M., Krobitsch S., Martowicz A., Gastl G., Spizzo G. (2011). Effects of EpCAM overexpression on human breast cancer cell lines. BMC Cancer.

[48] Wang X., Osada T., Wang Y., Yu L., Sakakura K., Katayama A., McCarthy J.B., Brufsky A., Chivukula M., Khoury T. (2010). CSPG4 protein as a new target for the antibody-based immunotherapy of triple-negative breast cancer. J. Natl. Cancer Inst..

[49] Jin L.L., Lu H.J., Shao J.K., Wang Y., Lu S.P., Huang B.F., Hu G.N., Jin H.C., Wang C.Q. (2024). Relevance and mechanism of STAT3/miR-221-3p/Fascin-1 axis in EGFR TKI resistance of triple-negative breast cancer. Mol. Cell Biochem..

[50] McKnight B.N., Kim S., Boerner J.L., Viola N.T. (2020). Cetuximab PET delineated changes in cellular distribution of EGFR upon dasatinib treatment in triple negative breast cancer. Breast Cancer Res..

[51] (n.d.), The Human Protein Atlas. ANK3 in Cancer. https://www.proteinatlas.org/ENSG00000151150-ANK3/cancer.

[52] Kurozumi S., Joseph C., Raafat S., Sonbul S., Kariri Y., Alsaeed S., Pigera M., Alsaleem M., Nolan C.C., Johnston S.J. (2019). Utility of ankyrin 3 as a prognostic marker in androgen-receptor-positive breast cancer. Breast Cancer Res. Treat..

[53] Alkaraki A., Alshaer W., Wehaibi S., Gharaibeh L., Abuarqoub D., Alqudah D.A., Al-Azzawi H., Zureigat H., Souleiman M., Awidi A. (2020). Enhancing chemosensitivity of wild-type and drug-resistant MDA-MB-231 triple-negative breast cancer cell line to doxorubicin by silencing of STAT 3, Notch-1, and beta-catenin genes. Breast Cancer.

[54] Jia T., Zhang L., Duan Y., Zhang M., Wang G., Zhang J., Zhao Z. (2014). The differential susceptibilities of MCF-7 and MDA-MB-231 cells to the cytotoxic effects of curcumin are associated with the PI3K/Akt-SKP2-Cip/Kips pathway. Cancer Cell Int..

[55] Vess A., Blache U., Leitner L., Kurz A.R.M., Ehrenpfordt A., Sixt M., Posern G. (2017). A dual phenotype of MDA-MB-468 cancer cells reveals mutual regulation of tensin3 and adhesion plasticity. J. Cell Sci..

[56] Albitar L., Pickett G., Morgan M., Wilken J.A., Maihle N.J., Leslie K.K. (2010). EGFR isoforms and gene regulation in human endometrial cancer cells. Mol. Cancer.

[57] Gellersen B., Wolf A., Kruse M., Schwenke M., Bamberger A.M. (2013). Human endometrial stromal cell-trophoblast interactions: Mutual stimulation of chemotactic migration and promigratory roles of cell surface molecules CD82 and CEACAM1. Biol. Reprod..

[58] Grimm A., Hussain A., Sheng W., Zhang C., Al-Rawe M., Meinhold-Heerlein I. (2022). Development and evaluation of antibody-fluorophore-conjugates to detect endometrial tissue. Geburtshilfe Und Frauenheilkd..

[59] O’Shannessy D.J., Somers E.B., Smale R., Fu Y.S. (2013). Expression of folate receptor-alpha (FRA) in gynecologic malignancies and its relationship to the tumor type. Int. J. Gynecol. Pathol..

[60] Wen K.C., Sung P.L., Chou Y.T., Pan C.M., Wang P.H., Lee O.K., Wu C.W. (2018). The role of EpCAM in tumor progression and the clinical prognosis of endometrial carcinoma. Gynecol. Oncol..

[61] Gronemeyer T., Godin G., Johnsson K. (2005). Adding value to fusion proteins through covalent labelling. Curr. Opin. Biotechnol..

[62] Sograte-Idrissi S., Schlichthaerle T., Duque-Afonso C.J., Alevra M., Strauss S., Moser T., Jungmann R., Rizzoli S.O., Opazo F. (2020). Correction: Circumvention of common labelling artefacts using secondary nanobodies. Nanoscale.

[63] Bales C.E. (2006). Laboratory techniques. Koss’s Diagnostic Cytology and Its Histopathologic Bases.

[64] Maier J., Traenkle B., Rothbauer U. (2015). Real-time analysis of epithelial-mesenchymal transition using fluorescent single-domain antibodies. Sci. Rep..

[65] Braun M.B., Traenkle B., Koch P.A., Emele F., Weiss F., Poetz O., Stehle T., Rothbauer U. (2016). Peptides in headlock--a novel high-affinity and versatile peptide-binding nanobody for proteomics and microscopy. Sci. Rep..

[66] Jung K.H., Kim S.F., Liu Y., Zhang X. (2019). A Fluorogenic AggTag Method Based on Halo- and SNAP-Tags to Simultaneously Detect Aggregation of Two Proteins in Live Cells. Chembiochem.

[67] Gautier A., Nakata E., Lukinavicius G., Tan K.T., Johnsson K. (2009). Selective cross-linking of interacting proteins using self-labeling tags. J. Am. Chem. Soc..

[68] Pellett P.A., Sun X., Gould T.J., Rothman J.E., Xu M.Q., Correa I.R., Bewersdorf J. (2011). Two-color STED microscopy in living cells. Biomed. Opt. Express.

