# Supplementary material for: Development of SNAP-Tag Based Nanobodies as Secondary Antibody Mimics for Indirect Immunofluorescence Assays
Source: Cells. 2025 May 10;14(10):691. doi: 10.3390/cells14100691 (PMC12110209; doi:10.3390/cells14100691)
Supplement: Supplementary file 1 [file cells-14-00691-s001.zip › cells-3513345-supplementary.pdf]

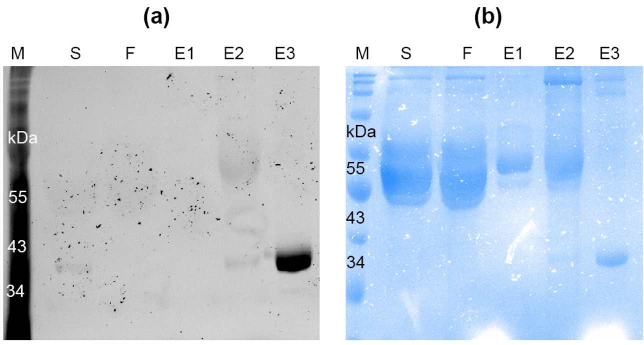

Figure S1. Enrichment of Nb anti-IgG1 Fc-SNAP. (**a**) All fractions were collected during enrichment, then conjugated to SNAP-Surface® Alexa Fluor® 488 followed SDS-PAGE running. (**b**) Visualization of the corresponding SDS-PAGE in Coomassie brilliant blue staining. SDS gel was imaged by ChemiDoc XRS+ System. M: Blue prestained protein standard broad range (11-250 kDa). S: filtered supernatant. F: flowthrough. E1: protein eluted in 10 mM imidazole. E2: protein eluted in 40 mM imidazole. E3: protein eluted in 250 mM imidazole.

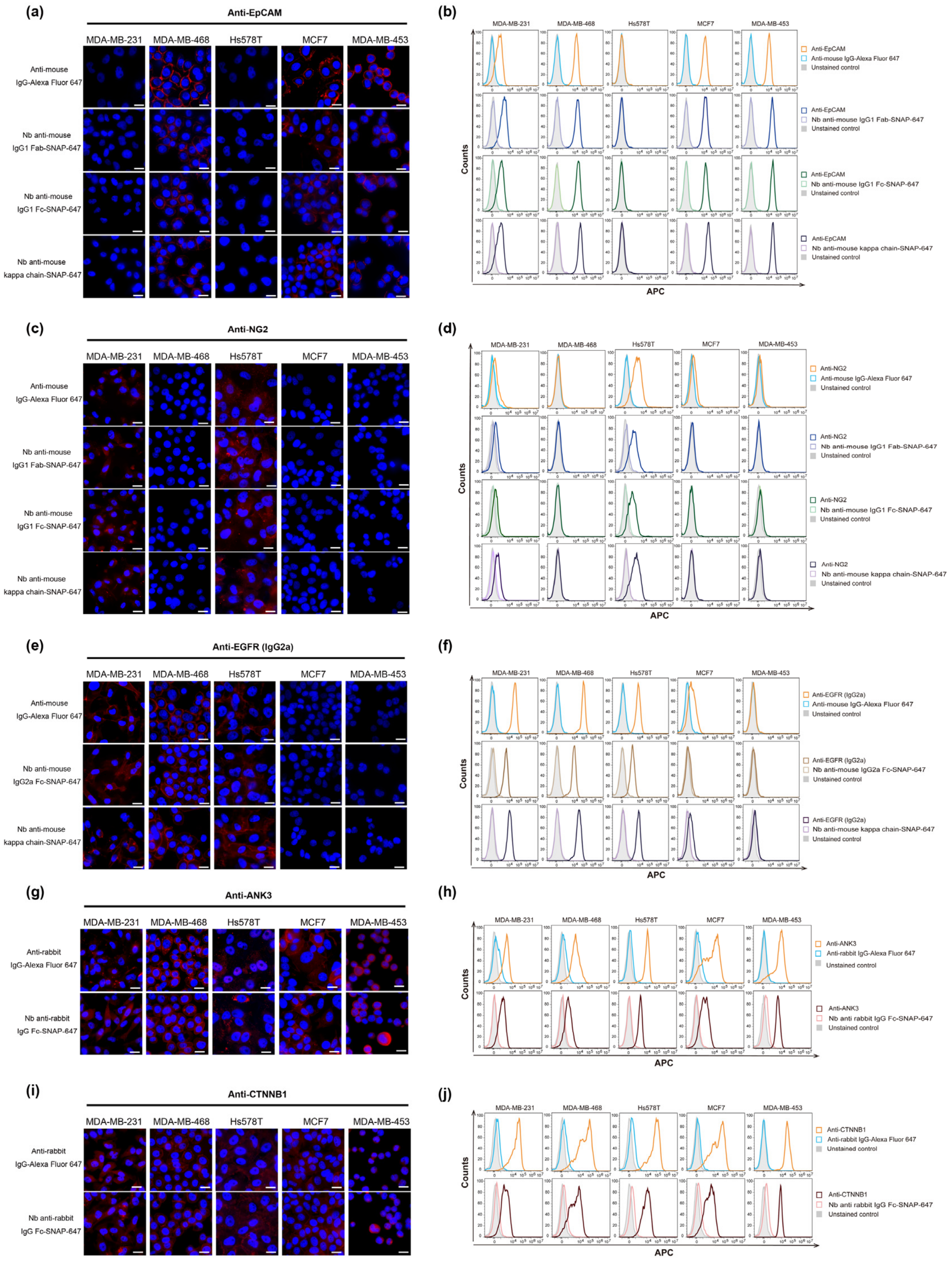

Figure S2. Fluorescence microscope (**a**, **c**, **e**, **g**, **i**) and flow cytometry analysis (**b**, **d**, **f**, **h**, **j**) with Nb anti-IgG-SNAP in breast cancer cell lines. Breast cells were incubated with anti-EpCAM, anti-NG2, anti-EGFR (IgG2a), anti-ANK3 and anti-CTNNB1, then they were detected with Surface®Alexa Fluor® 647-labeled Nbs anti-IgG-SNAP or corresponding commercial antibodies. The red signal presented Alexa Fluor® 647 channel, blue signal was nuclear counterstain with Hochest 33342 (scale bar 20 μm).

Table S1. Different antigens expressed levels in breast cancer cell lines.

Target	MDA-MB-231	MDA-MB-468	Hs578T	MCF-7	MDA-MB-453
EpCAM	low	medium	low	high	high
NG2	medium	low	high	low	low
EGFR	high	high	medium	low	low
ANK3	medium	medium	high	medium	high
CTNNB1	medium	medium	high	medium	high

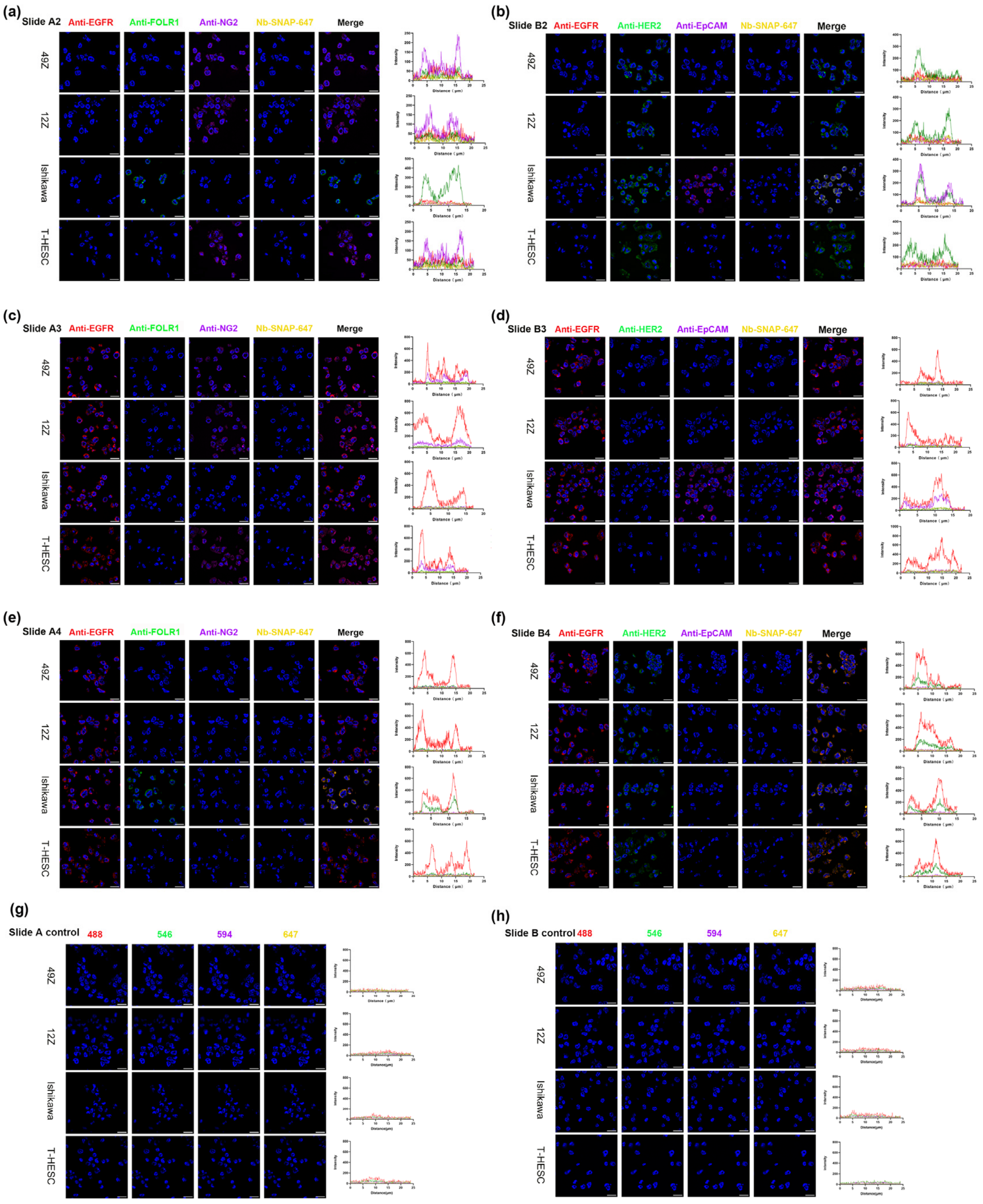

Figure S3. The multicolor immunofluorescence and fluorescence intensity profiles of leave-one-out control strategy and Nbs anti-IgG-SNAP-647 post-incubation. (**a**) No anti-$EGFR^{mouse}$ (red)staining on slide A2. (**b**) No anti-$EGFR^{rabbit}$ (red) staining on slide B2. (**c**) No anti-FOLR1 staining (green) on slide A3. (**d**) No anti-HER2 staining (green) on slide B3. (**e**) No anti-NG2 staining (magenta) on slide A4. (**f**) No anti-EpCAM staining (magenta) on slide B4. (**g**) Only Nbs anti-IgG-SNAP-Alexa Fluor dyes stained with slide A control. (**h**) Only Nbs anti-IgG-SNAP-Alexa Fluor dyes stained with slide B control. Fluorescence intensity profiles of SNAP-Surface® Alexa Fluor® 488 (red curve), SNAP-Surface® Alexa Fluor® 546 (green curve), SNAP-Surface® Alexa Fluor® 594 (magenta curve) and SNAP-Surface® Alexa Fluor® 647 (yellow curve). Magnification is indicated by the white scale bars (20 μm).
